# Therapeutic Effects of Omentin-1 on Pulmonary Fibrosis by Attenuating Fibroblast Activation via AMP-Activated Protein Kinase Pathway

**DOI:** 10.3390/biomedicines10112715

**Published:** 2022-10-26

**Authors:** Yan Zhou, Yunna Zhang, Haipeng Cheng, Xiaohong Li, Dandan Feng, Shaojie Yue, Jianping Xu, Hui Xie, Ziqiang Luo

**Affiliations:** 1 Department of Physiology, Xiangya School of Medicine, Central South University, Changsha 410013, China; 2 Department of Pediatrics, Xiangya Hospital, Central South University, Changsha 410008, China; 3 Department of Orthopedics, Xiangya Hospital, Central South University, Changsha 410008, China; 4 Hunan Key Laboratory of Organ Fibrosis, Changsha 410008, China

**Keywords:** bleomycin, lung fibrosis, omentin-1, fibroblast activation, myofibroblast, AMPK pathway

## Abstract

Idiopathic pulmonary fibrosis (IPF) is a fatal age-related chronic lung disease, characterized by progressive scarring of the lungs by activated fibroblasts. The effect of omentin-1 against pulmonary fibrosis and fibroblast activation has not been investigated. The purpose of this experiment is to investigate the role of omentin-1 in bleomycin (BLM)-induced lung fibrosis and its mechanism. Our results showed that the loss of omentin-1 exaggerated lung fibrosis induced by BLM. On the contrary, adenoviral-overexpression of omentin-1 significantly alleviated BLM-induced lung fibrosis both in preventive and therapeutic regimens. Moreover, omentin-1 prevented fibroblast activation determined by a decreased number of S100A4^+^ (fibroblasts marker) α-SMA^+^ cells in vivo, and a decreased level of α-SMA expression both in mice primary fibroblasts and human primary fibroblasts induced by TGF-β in vitro. Furthermore, the phosphorylation of AMP-activated protein kinase (p-AMPK) was significantly lower in the fibrotic foci induced by BLM, and the adenoviral-overexpression of omentin-1 significantly increased the p-AMPK level in vivo. Importantly, Compound C, the inhibitor of AMPK, significantly attenuated the protective effect of omentin-1 on BLM-induced lung fibrosis and reversed the effect of omentin-1 on fibroblast activation by TGF-β. Omentin-1 can be a promising therapeutic agent for the prevention and treatment of lung fibrosis.

## 1. Background

Idiopathic pulmonary fibrosis (IPF) is an age-related chronic disease characterized by a progressive scarring of the lungs, the remodeling of fibrotic tissue, and collagen deposition, as well as reduced lung compliance and gas exchange, finally leading to respiratory failure and death [1]. In recent years, the incidence of IPF has been gradually increasing, reaching 2.8–18 cases per 100,000 people per year. The median survival time is 2–4 years after diagnosis [1]. Although recent progresses have been made in the study of the pathogenesis of pulmonary fibrosis, the two drugs approved by the Food and Drug Administration (FDA), nintedanib and pirfenidone, can only slow down the disease process but fail to effectively improve prognosis [2,3]. Despite therapeutic advancements, lung architecture and function cannot be restored with the currently available treatments, and organ transplantation often remains the only option [4]. Therefore, the investigation of the cellular and molecular mechanisms underlying pulmonary fibrosis and the search of novel therapeutic strategies are desperately needed.

In the initial stages of lung injury, a large number of fibroblasts proliferate and differentiate into myofibroblasts, which are responsible for the production of collagen I, collagen III, fibronectin, laminin and other extracellular matrix components, as well as for the deposition and remodeling of extracellular matrix (ECM) proteins [5,6]. During the resolution of the normal repair program, myofibroblasts are eliminated by apoptosis or dedifferentiated into low-activity fibroblasts to restore normal tissue homeostasis [7]. However, under pathological conditions, myofibroblasts are maintained in an active state and escape apoptosis, resulting in higher extracellular matrix deposition and fibrosis [5]. In this regard, myofibroblasts are known as effector cells in pulmonary fibrosis and other fibrotic diseases. Inhibiting the differentiation of fibroblasts into myofibroblasts or inducing their apoptosis has the potential to halt the development of pulmonary fibrosis and even reverse the fibrotic process [5].

Omentin-1 (also known as intelectin-1) is an adipokine of 313 amino acids, first reported in 2005 [8]. Omentin-1 is mainly expressed in human omental adipose tissue, subcutaneous adipose tissue, as well as the small intestine. Lung, heart, ovary, placenta, skeletal muscle and kidneys also express low levels of omentin-1 [8,9]. Recently, omentin-1 was found to inhibit the production of reactive oxygen species (ROS) and apoptosis induced by doxorubicin [10]. In addition, it promotes the proliferation and inhibits the apoptosis of mesenchymal stem cells [11]. Omentin-1 also inhibits the proliferation of human aortic smooth muscle cells and the expression of type I and type III collagen, and reduces the occurrence of atherosclerosis [12]. In the lung tissue, overexpression omentin-1 or exogenous recombinant omentin-1 was reported to reduce the endotoxin-induced increase of pro-inflammatory factors, such as IL-6 and TNF-α, and the degree of acute lung injury [13]. Omentin-1 is closely related to obesity, diabetes, insulin resistance, energy metabolism, inflammation, cancer, cardiovascular disease, as well as bone metabolism [14].

AMPK is a cell bioenergy sensor and metabolic regulator, monitoring changes in the level of Adenosine Triphosphate (ATP) in cells. The activation of AMPK can enhance ATP production and/or decrease the rate of ATP-utilizing pathways during the metabolic process [15]. Mammalian AMPK is a heterotrimeric complex, containing α, β and γ subunit. The activity of AMPK is regulated by phosphorylation of threonine 172 (T172) of the α subunit [16]. Research showed that the threonine phosphorylation level at position 172 of AMPK and the enzyme activity of myofibroblasts in IPF patients with fibrosis are decreased. Metformin can enhance AMPK activity and inhibit the differentiation of myofibroblasts induced by TGF-β1 [17,18]. Moreover, several drugs that activate AMPK pathways in fibroblasts and lung fibrosis tissue significantly attenuate lung fibrosis [19,20,21]. These studies indicate that activating AMPK is a new target of anti-pulmonary fibrosis. According to the previous literature, the anti-inflammation, myocardial protection and anti-atherosclerosis effects of omentin-1 depend on the mediation of the AMP-activated protein kinase (AMPK) [22,23,24,25].

We have previously shown that adenovirus-driven omentin-1 overexpression alleviates lung injury and inflammation induced by bleomycin (BLM) by preventing macrophage activation [26], however its impact on lung fibrosis has not been yet investigated. Although studies have shown that the use of anti-inflammatory drugs during acute lung injury can reduce the degree of pulmonary fibrosis in mice caused by BLM [27], drugs with anti-inflammatory effects alone have no definite efficacy on patients with idiopathic pulmonary fibrosis [28]. Besides the anti-inflammatory effect of omentin-1, it is unclear whether omentin -1 has a direct anti-fibrosis effect. Our study aimed to uncover the possible beneficial effects of omentin-1 in BLM-induced fibrosis, specifically focusing on its impact on direct anti-fibrosis and fibroblast activation.

## 2. Methods

### 2.1. Experimental Animals and Treatment

The experiments were conducted on eight-week-old male C57BL/6 mice (specific-pathogen-free [SPF] grade. All mice were maintained under pathogen-free conditions and provided with standard food and water. The body weight was recorded every day. The mice were anesthetized with sodium pentobarbital (80 mg kg^−1^, intraperitoneal injection) and every effort was made to minimize animal suffering. The mice were intratracheally injected with 50 μL of BLM (1.25 mg/kg, Nippon Kayaku, Tokyo, Japan) to induce pulmonary fibrosis, or with sterile PBS as a control. Adenovirus-omentin-1 (Ad-omentin-1) or control adenovirus constructs were injected at 5 × 10^7^ PFU per mouse into the tail vein 3 days before and 4 days after the BLM challenge, for the preventive treatment, and 11 days and 18 days after the BLM challenge for the therapeutic treatment. AMPK inhibitor, Compound C (10 mg/kg, topscience, Shanghai, China) was administrated at 14 days after the BLM challenge in therapeutic design. Omentin-1-deficient (omentin-1^−/−^) mice were fed as previously described; Omentin-1-deficient (omentin-1^−/−^) mice C57BL/6 mice were generated by Cyagen Biosciences Inc. (Guangzhou, China) using TALEN technology. TALEN target sites were designed in exon 4 of the mouse omentin-1 gene and one base (A) was inserted into the sequence of omentin-1 gene in one strand, causing a frameshift mutation and an early stop of omentin-1 protein translation [29]. Eight-week-old male omentin-1^−/−^ mice and their WT male littermates were used in the relevant experiments. The mice were intratracheally injected with 50 μL of BLM (1.0 mg/kg), or sterile PBS as a control, after being anesthetized. Lung samples were collected on day 7, 14, 21 or 28 after BLM administration and used for the experiments or stored at −80 °C until analysis.

### 2.2. Histopathology

The lungs were isolated and fixed in 4% paraformaldehyde and embedded in paraffin. Sections were stained with hematoxylin and eosin (H&E) and Masson’s trichrome. Ashcroft fibrosis score was used for grading fibrosis scale.

### 2.3. Fibroblasts Isolation and Culture

Human lung fibroblasts (HLF) were purchased from ATCC, cultured in DMEM (Hyclone, Rockford, IL, USA) containing 10% FBS (CellMax, Lanzhou, China) in a humified CO_2_ incubator at 37 °C.

Primary fibroblasts were isolated and purified from newborn 3–7-day-old C57BL/6 mice, as previously described [30]. In brief, the lungs were isolated under sterile conditions. The lung tissue was finely chopped and digested with collagenase I (1 mg/mL, Sigma-Aldrich, St. Louis, MO, USA) and DNase I (10 μg/mL, Solarbio, Beijing, China) for 1 h at 37 °C. Next, the cells were incubated in DMEM/F-12 (Hyclone, Rockford, IL, USA) containing 10% FBS (CellMax, Lanzhou, China), 1% penicillin-streptomycin mixture (Solarbio, Beijing, China) in a humified CO_2_ incubator at 37 °C for several passages. The cells were used at passage 3–6.

For omentin-1 pretreatment experiment, HLFs and Primary mouse lung fibroblasts were cultured in medium containing 10% FBS, and then the medium was replaced for 12 h with FBS-free for cell starvation. The cells were pretreated for 1 h with recombinant omentin-1 (500 ng/mL or 1000 ng/mL, Prospec, Ness-Ziona, Israel), and fibroblasts were activated with human or mouse TGF-β1 (10 ng/mL, R&D, Minneapolis, CA, USA) for 48 h. In some experiments, Compound C (10 μM) was added for 48 h to show the effect of inhibition of the AMPK pathway.

### 2.4. RNA Isolation and Quantitative Polymerase Chain Reaction (qPCR)

Total RNA was isolated with TRIzol reagent (Takara, Kyoto, Japan) from lung tissues and fibroblasts according to the manufacturer’s protocol. Complementary DNA (cDNA) was transcribed from 1 μg of total RNA with a First Strand cDNA Synthesis Kit (Takara, Kyoto, Japan). Real-time PCR was performed using SYBR Green signals (Bio-Rad, CA, USA), and detected with a Bio-Rad real-time PCR system (CFX96 Touch™, Bio-Rad, USA). The relative expression mRNA level was determined after normalization to glyceraldehyde-3-phosphate dehydrogenase (*GAPDH*) gene or β2 microglobulin (B2M) by using the formula 2^−ΔΔCT^. The primer sequences are shown in Table 1.

### 2.5. Western Blotting

For Western blot analysis, lung tissue was homogenized in ice-cold RIPA lysis buffer (Beyotime Biotechnology, Shanghai, China) containing a proteinase inhibitor cocktail (Roche Diagnostics, Indianapolis, IN, USA) to harvest total proteins. Total protein concentration was measured using a BCA kit (Thermo Scientific, MA, USA). Next, 30 μg of total proteins were separated by 10% SDS-PAGE and transferred to polyvinylidene fluoride (PVDF) membranes (Millipore, Burlington, MA, USA). After blocking with 5% fat-free milk for 1 h, the membranes were incubated with the primary antibody overnight at 4 °C, followed by incubation with the appropriate horseradish-peroxidase (HRP)-conjugated secondary antibodies (1:7500; Abcam, Cambridge, UK). Images were obtained using a ChemiDoc XRS system (Bio-Rad) with enhanced chemiluminescence reagents (Millipore, MA, USA). The relative density was estimated by using Image Lab analysis software, and protein levels were normalized to β-tubulin or GAPDH. The primary antibodies were as follows: anti-β-tubulin (1:1000; Servicebio, Wuhan, China), anti-GAPDH (1:2000; Servicebio, Wuhan, China), anti-α smooth muscle actin (α-SMA) (1:1000; Servicebio, Wuhan, China), anti-collagen III (Col3) (1:1000; Servicebio, Wuhan, China), and anti-omentin-1 (1:1000; R&D, CA, USA).

### 2.6. Hydroxyproline Assay

The collagen content in lung tissue was detected by using hydroxyproline (HYP) assay kits (Jiancheng Biotechnology Institute, Nanjing, China) according to the manufacturer’s protocol. The absorbance at a wavelength of 550 nm was measured by using a microplate reader (Thermo Fisher Scientific, Waltham, MA, USA).

### 2.7. Immunohistochemistry

Mice lung tissue was fixed, embedded in paraffin, sectioned to 5-μm-thick slices, deparaffinized, and dehydrated. Endogenous peroxidase activity was quenched in 0.3% H_2_O_2_ for 10 min, and the slides were blocked with 5% bovine serum albumin (BSA) for 30 min. The sections were stained with primary antibodies against α-SMA (1:100; Servicebio, Wuhan, China), collagen I (1:100; Servicebio, Wuhan, China) or collagen III (1:100; Servicebio, Wuhan, China) overnight at 4 °C, followed by incubation with an HRP-conjugated secondary antibody (1:100; Sigma-Aldrich, MI, USA) at room temperature (20–25 °C) for 30 min. Images were acquired by a microscope (Nikon, Tokyo, Japan).

### 2.8. Enzyme-Linked Immunosorbent Assay

ELISA was used to determine omentin-1 levels in lung homogenates. The lung tissues were homogenized in PBS containing protease inhibitors (Thermo Fisher Scientific, MA, USA) and centrifuged at 10,000× *g* for 20 min to remove insoluble debris. The supernatants were assayed with anti-mouse omentin-1 ELISA kits (CUSABIO, Wuhan, China).

### 2.9. Immunofluorescence Staining

Paraffin-embedded lung tissue was deparaffinized and dehydrated for immunofluorescence staining. The slides were blocked with 5% BSA for 30 min. Then, the sections were stained overnight at 4 °C with mouse anti-α-SMA antibodies (1:100; Servicebio, Wuhan, China) and rabbit anti-S100A4 (1:100; Servicebio, Wuhan, China), followed by staining with Alexa Fluor 488-goat anti-mouse secondary antibodies (1:100, Beyotime, Shanghai, China) and Alexa Fluor 594-goat anti-rabbit secondary antibodies (1:100, Beyotime, Shanghai, China) or anti-p-APMK antibodies (1:100; Elabscience, Wuhan, China), followed by staining with Alexa Fluor 594-goat anti-rabbit secondary antibodies (1:100, Beyotime, Shanghai, China) at room temperature for 30 min. For immunofluorescence staining of fibroblasts, the cells were grown on coverslips and subjected to different treatments. The cells were fixed with 4% paraformaldehyde for 15 min and permeabilized with Triton X-100 (0.025%, Solarbio, Beijing, China). The cells were blocked with 5% BSA for 30 min, and stained overnight at 4 °C with the primary antibodies, i.e., mouse anti-α-SMA (1:100; Servicebio, Wuhan, China) and mouse anti- collagen III (1:100; Servicebio, Wuhan, China), followed by staining with Alexa Fluor 594-goat anti-mouse secondary antibodies (1:100, Beyotime, Shanghai, China) at room temperature for 30 min. Moreover, 4′,6-diamidino-2-phenylindole (DAPI; Proteintech, Wuhan, China) was used to stain the cell nuclei. The images were acquired by a fluorescence microscope (Nikon, Tokyo, Japan).

### 2.10. Flow Cytometry for Detecting of α-SMA Level

To determine the α-SMA protein level in fibroblasts, cells after treatment were resuspended, fixed and permeabilized, and stained with FITC Anti-alpha smooth muscle Actin antibody (Abcam, Cambridge, UK) for 45 min in the dark on ice. The fluorescence intensity of α-SMA was detected by a flow cytometer (BD Bioscience, Franklin Lakes, NJ, USA).

### 2.11. Statistical Analysis

Data were expressed as the mean ± SD. All experiments were repeated at least three times. Two-group comparisons were statistically analyzed by Student’s *t*-test. Comparisons between multiple groups were analyzed by one-way analysis of variance followed by Student-Newman-Keuls (SNK) test. The threshold for statistical significance was set at *p* < 0.05. GraphPad Prism 8 was used for statistical analysis.

## 3. Results

### 3.1. Omentin-1 Expression Increases during the Late Stages of Lung Fibrosis in Experimental Models

**Figure 1:**
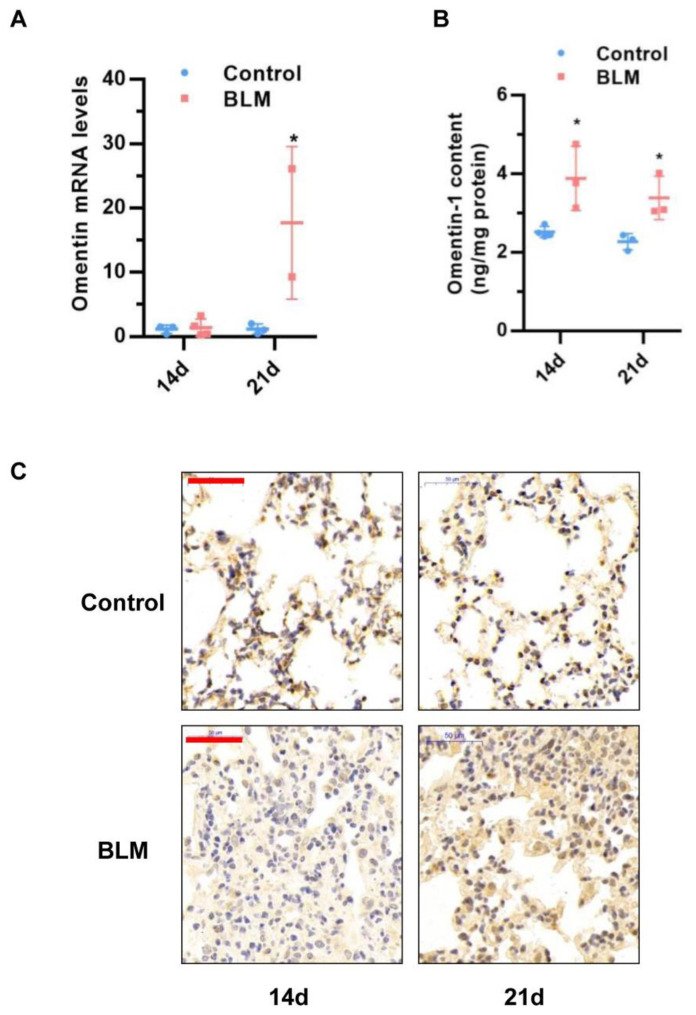
The expression level of omentin-1 in mouse lung tissue after Bleomycin (BLM) challenge for 14d and 21d. (**A**) The expression of omentin-1 mRNA in the lung tissue of mice detected by qPCR (*n* = 3); (**B**) The content of omentin-1 protein in lung homogenate of mice detected by ELISA (*n* = 4). (**C**) Immunohistochemical staining of lung sections for omentin-1 (Bar = 50 μm, *n* = 4). The data are presented as the mean ± SD. * *p* < 0.05 vs. control group.

Our previous study showed that omentin-1 levels decrease during the inflammatory phase of the bleomycin-induced lung injury model at days 3–7 [26]. To further examine omentin-1 expression in this model, we first examined the mRNA and protein levels of omentin-1 in lung tissue by real time PCR and ELISA at day 14 and 21 after the bleomycin challenge. Results showed that the mRNA level of omentin-1 was significantly increased at 21 days after BLM exposure (Figure 1A), while the protein contents significantly increased at 14 and 21 days after the BLM challenge, as determined by ELISA (Figure 1B) and Immunochemistry (Figure 1C). These results demonstrated that omentin-1 expression changed in mice with BLM-induced fibrosis, as both mRNA and protein levels were significantly increased at 21 days after BLM exposure.

### 3.2. Omentin-1-Deficient Mice Show Exaggerated Lung Fibrotic Responses

**Figure 2:**
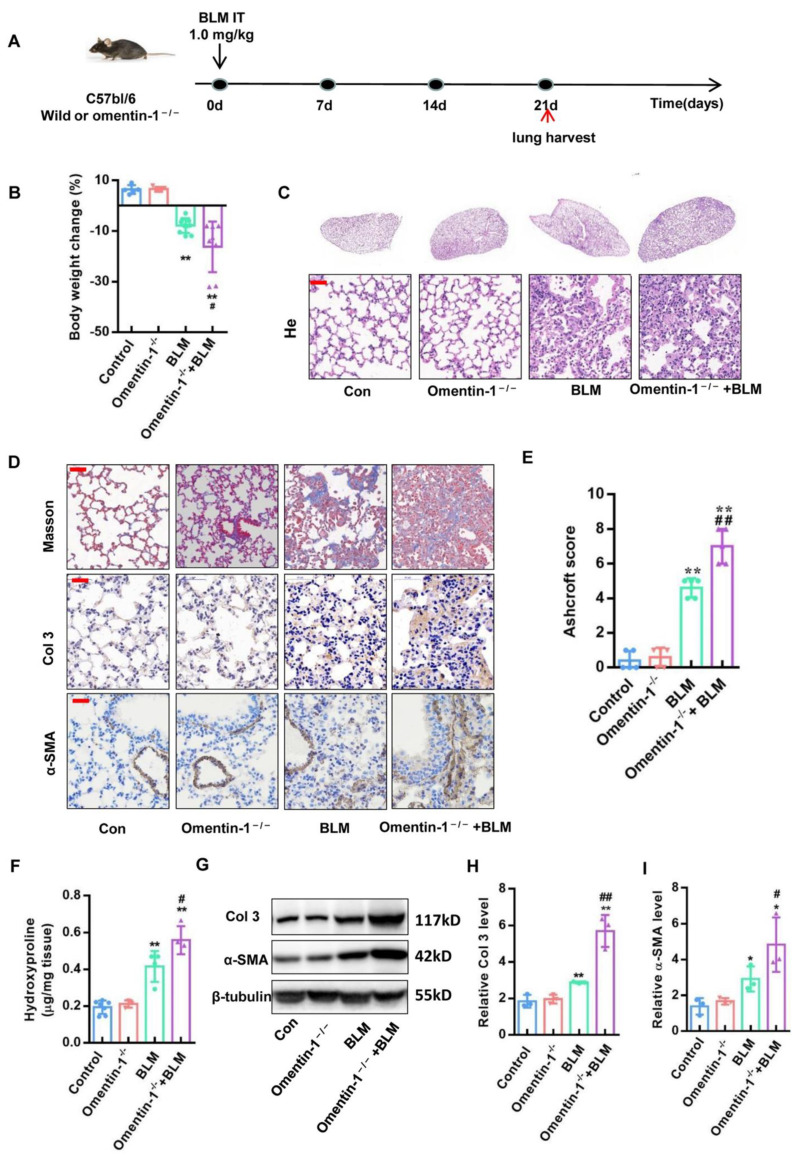
The effect of omentin-1 deficiency on BLM-induced lung fibrosis at 21 d. (**A**) Experimental design: BLM (1.0 mg/kg) was injected to induce fibrosis (21 days) in wild-type and Omentin-1^−/−^ mice. (**B**) Weight change of mice 21 days after BLM challenge (*n* = 10). (**C**) Representative micrographs of H&E staining of lung tissue (Bar = 50 μm, *n* = 5). (**D**) Representative micrographs of Masson trichrome staining, immunohistochemical staining of lung sections for Collagen III (Col3) and α-SMA (Bar = 50 μm, *n* = 5). (E) Ashcroft score to grade fibrosis scale (*n* = 5). (**F**) Collagen content was measured by hydroxyproline (HYP) assay (*n* = 5). (**G**) The content of collagen III (Col3) and α-SMA in lung tissue detected by Western blotting with cropping gel at the relative molecular size. (*n* = 3). (**H**) Semi-quantitative analysis of Col3 level evaluated by Western blotting. (**I**) Semi-quantitative analysis of α-SMA level evaluated by Western blotting. The data are presented as the mean ± SD. * *p* < 0.05, ** *p* < 0.01 vs. control group. # *p* < 0.05, ## *p* < 0.01 vs. BLM group.

Omentin-1 deletion was generated by the successful insertion of an adenine base into the sequence of omentin-1 gene, and by the absence of omentin-1 protein expression in the small intestine (the organ showing the highest expression of omentin-1 in mice) of omentin-1^−/−^ mice [29]. Omentin-1^−/−^ mice and wild-type littermates (WT) were challenged with BLM (1.0 mg/kg) or PBS via intratracheal instillation for 21 days (Figure 2A). Compared with the control group, the body weight of omentin-1^−/−^ mice have no obvious change. The body weight of wild-type mice significantly decreased after the BLM challenge (Figure 2B), while the body weight of omentin-1^−/−^ mice further decreased after injury by BLM compared with wild-type mice (Figure 2B). These results showed that the loss of omentin-1 could accelerate the body weight loss of BLM-induced pulmonary fibrosis mice. H&E staining showed that BLM caused thickening of the alveolar septum and the alveolar collapsed, while loss of omentin-1 significantly accelerated the morphological structure damage of lung tissue caused by BLM (Figure 2C). Moreover, Masson staining was used to observe the collagen deposition in the lung tissue of mice. Results showed that there is higher level of collagen stained as blue in the lung tissue of omentin-1^−/−^ mice challenged by BLM, compared to WT mice challenged by BLM (Figure 2D). Moreover, the Ashcroft score, which can grade lung fibrosis scale, showed that the score significantly increased after the BLM challenge and was much higher in BLM-challenged omentin-1^−/−^ mice compared to BLM-induced WT mice (Figure 2E). Hydroxyproline (HYP) is a specific component of collagen, and measuring the content of HYP in lung tissue can indirectly reflect the content of collagen. The results showed that HYP content was much higher in BLM-challenged omentin-1^−/−^ mice compared to BLM-induced WT mice (Figure 2F). Furthermore, Collagen Ⅲ is an important component of collagen deposition in pulmonary fibrosis [31]. Immunochemistry staining and Western blotting confirmed increased collagen III (Col3) expression in the lung tissue of BLM-induced omentin-1^−/−^ mice compared to BLM-induced WT mice (Figure 2D,G,H). In response to tissue injury, fibroblasts can differentiate into myofibroblasts, which is the effector fibrogenic cells characterized by de novo synthesis of the alpha smooth muscle actin (α-SMA) and stress fiber formation [32,33]. Immunochemistry staining and Western blotting analysis showed increased α-SMA levels in BLM-treated omentin-1^−/−^ mice compared to WT mice (Figure 2D,G,I). Taken together, our results show an exaggerated fibrotic response to BLM in omentin-1^−/−^ mice, suggesting an anti-fibrotic role for omentin-1 in vivo in the bleomycin-induced lung fibrosis model.

### 3.3. Omentin-1 Overexpression Attenuates BLM-Induced Lung Fibrosis with Preventative and Therapeutic Treatment

**Figure 3:**
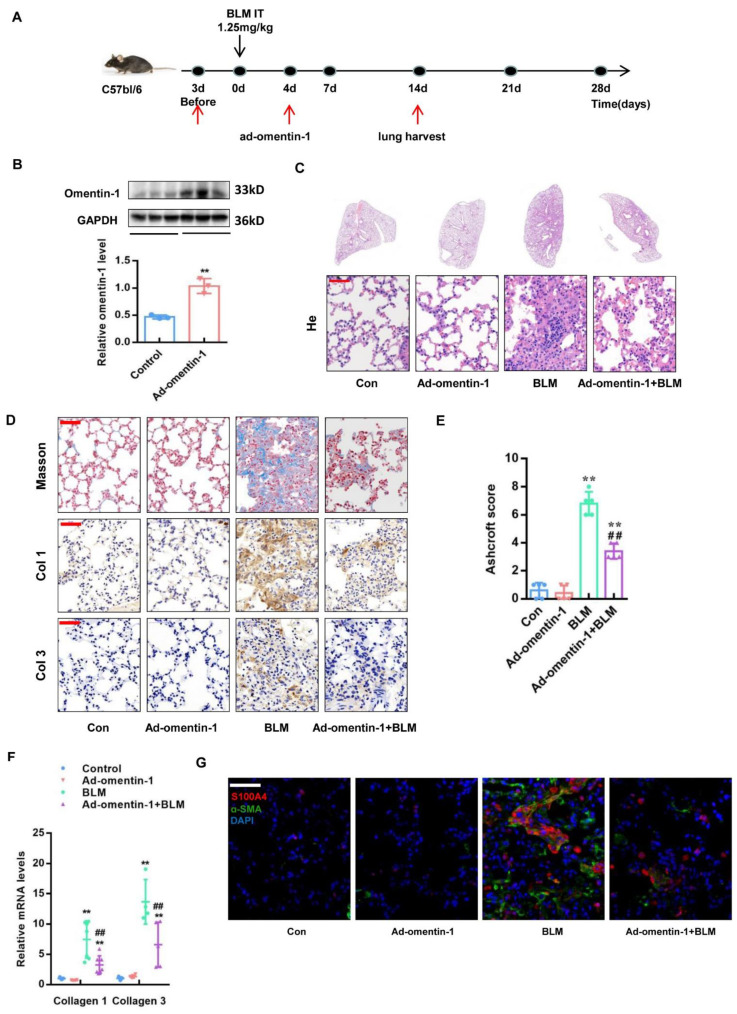
The effect of omentin-1 on BLM-injured mice by preventative treatment. (**A**) Experimental design: The preventive treatment with ad-omentin-1 was performed on D3 before BLM and D4 after BLM. BLM (1.25 mg/kg) was injected to induce fibrosis in wild-type mice; (**B**)The content of omentin-1 in lung tissue detected by western blotting with cropping gel at the relative molecular size (*n* = 3); (**C**) Representative micrographs of H&E staining of lung tissue (Bar = 50 μm, *n* = 5). (**D**) Representative micrographs of Masson trichrome staining, immunohistochemical staining of lung sections for collagen I (Col1) and Col3 (Bar = 50 μm, *n* = 5). (**E**) Ashcroft score to grade fibrosis scale (*n* = 5). (**F**) The expression of Col1 and Col3 mRNA in the lung tissue of mice detected by qPCR (*n* = 4). (**G**) The expression and localization of α-SMA and S100A4 in lung tissue detected by immunofluorescence (bar = 50 μm, *n* = 5). The data are presented as the mean ± SD. ** *p* < 0.01 vs. control group. ## *p* < 0.01 vs. BLM group.

We have previously shown that adenovirus-driven omentin-1 overexpression alleviates lung injury and inflammation induced by bleomycin (BLM) [26], however the direct impact of lung fibrosis has not been yet investigated. To further evaluating the effect of omentin-1 on lung fibrosis, adenoviral vectors containing omentin-1 (ad-omentin-1) or the control adenovirus were injected via the tail vein of mice at 3 days prior and 4 days after BLM airway installation for preventional treatment (Figure 3A). Omentin-1 overexpression in the lung tissue was validated by Western blotting at day 17 after adenovirus injection (day 14 after BLM insult), showing the protein level of omentin-1 increased in the lung tissue after ad-omentin-1 injection (Figure 3B). As demonstrated by H&E staining, preventional adenoviral overexpression of omentin-1 attenuated the destruction of lung tissue induced by BLM (Figure 3C). Masson staining showed that preventional omentin-1 overexpression reduced collagen deposition (blue staining) in lung tissue after the BLM challenge (Figure 3D). Moreover, the Ashcroft score, which can grade the lung fibrosis scale, showed that the score significantly increased after the BLM challenge and decreased by overexpression of omentin-1 (Figure 3E). Moreover, Immunochemistry staining (Figure 3D) and qPCR (Figure 3F) confirmed that omentin-1 overexpression significantly decreased the protein and mRNA level of collagen I (Col1) and Col3 in the lung tissue after BLM insult. These results demonstrated that preventative omentin-1 overexpression attenuated lung fibrosis induced by BLM.

**Figure 4:**
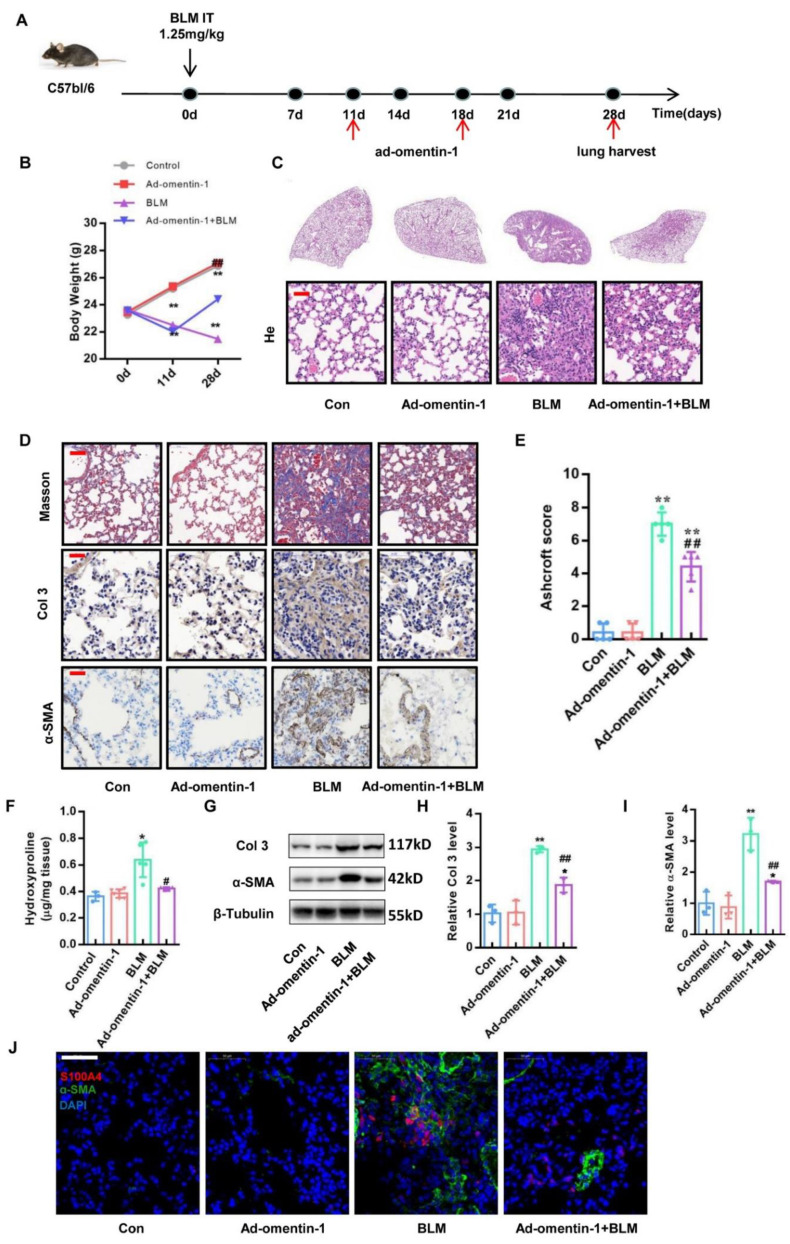
The effect of omentin-1 on BLM-injured mice by therapeutic treatment. (**A**) Experimental design: The therapeutic treatment with ad-omentin-1 was performed on D11 and D18 after BLM (fibrosis established). BLM (1.25 mg/kg) was injected to induce fibrosis in wild-type mice; (**B**) Body weight change of mice after BLM challenge (*n* = 10); (**C**) Representative micrographs of H&E staining of lung tissue (Bar = 50 μm, *n* = 5). (**D**) Representative micrographs of Masson trichrome staining, immunohistochemical staining of lung sections for Col3 and α-SMA (Bar = 50 μm, *n* = 5); (**E**) Ashcroft score to grade fibrosis scale (*n* = 5). (**F**) Collagen content was measured by hydroxyproline (HYP) assay (*n* = 5). (**G**)The content of Col3 and α-SMA in lung tissue detected by Western blotting with cropping gel at the relative molecular size (*n* = 3); (**H**) Semi-quantitative analysis of Col3 level evaluated by Western blotting; (**I**) Semi-quantitative analysis of α-SMA level evaluated by western blotting; (**J**) The expression and localization of α-SMA and S100A4 in lung tissue detected by immunofluorescence (bar = 50 μm, *n* = 5). The data are presented as the mean ± SD. * *p* < 0.05, ** *p* < 0.01 vs. control group. # *p* < 0.05, ## *p* < 0.01 vs. BLM group.

Since omentin-1 overexpression prevents inflammatory responses in the early phases of BLM-induced lung injury [26], whether omentin-1 prevents lung fibrosis through inhibition of inflammation or fibrotic response is unclear. We designed a therapeutic treatment in order to determine the direct anti-fibrosis effect of omentin-1 on established lung fibrosis. In this therapeutic regimen, mice were intratracheally challenged with BLM or PBS (control). Ad-omentin-1 or the control adenovirus were injected into the tail vein of mice at days 11 and 18 after the BLM challenge, while the fibrosis was established. The level of fibrosis was evaluated at 28 days (Figure 4A). The results of the body weight change in mice showed that the body weight in the BLM group significantly decreased after 11 days after the BLM insult. On the 28th day after the BLM injection, the body weight of mice in the BLM group continued to decrease compared with that on the 11th day. On the contrary, the overexpression of omentin-1 in the therapeutic regimen effectively promoted the body weight recovery of the BLM-injured mice (Figure 4B). H&E staining showed the alveolar septum thickening and the alveolar collapse in the lung tissue of the BLM group. Overexpression of omentin-1 could significantly improve BLM-induced thickening of alveolar septum and alveolar collapse (Figure 4C). Masson staining showed that the therapeutic overexpression of omentin-1 significantly reduced collagen deposition (blue staining) in lung tissue after the BLM challenge for 28 days (Figure 4D). Moreover, the Ashcroft score, which can grade the lung fibrosis scale, showed that the score significantly increased after the BLM challenge and decreased by the overexpression of omentin-1 (Figure 4E). The results showed that increased HYP content in the lung tissue of BLM-challenged mice was significantly decreased by the overexpression of omentin-1 (Figure 4F). Furthermore, immunochemistry staining and Western blotting confirmed that the increased protein level of Col3 in the lung tissue of BLM-injured mice were significantly attenuated by the overexpression of omentin-1 (Figure 4D,G,H). The therapeutic overexpression of omentin-1 also decreased the protein level of α-SMA induced by the BLM in lung tissue (Figure 4D,G,I).

To further explore the cellular mechanism of omentin-1 in alleviating pulmonary fibrosis, we used immunofluorescence to detect fibroblast activation in lung tissue. Fibroblasts activated or differentiated to myofibroblasts were detected as double positive cells for S100A4 (a fibroblast marker) and α-SMA (a myofibroblast marker) [34,35]. The results showed that, both in preventive and therapeutic regimens, omentin-1 overexpression significantly decreased the level of α-SMA (green) in S100A4^+^ cells (red) (Figure 3G and Figure 4J), indicating that omentin-1 prevented fibroblast activation in vivo. These results indicate that the overexpression of omentin-1 not only exerts an anti-inflammation effect as showed in our previous study [26], but also plays a direct anti-fibrosis role in lung fibrosis induced by BLM by preventing fibroblast activation.

### 3.4. Recombinant Omentin-1 Attenuates TGF-β-Induced Myofibroblast Differentiation In Vitro

**Figure 5:**
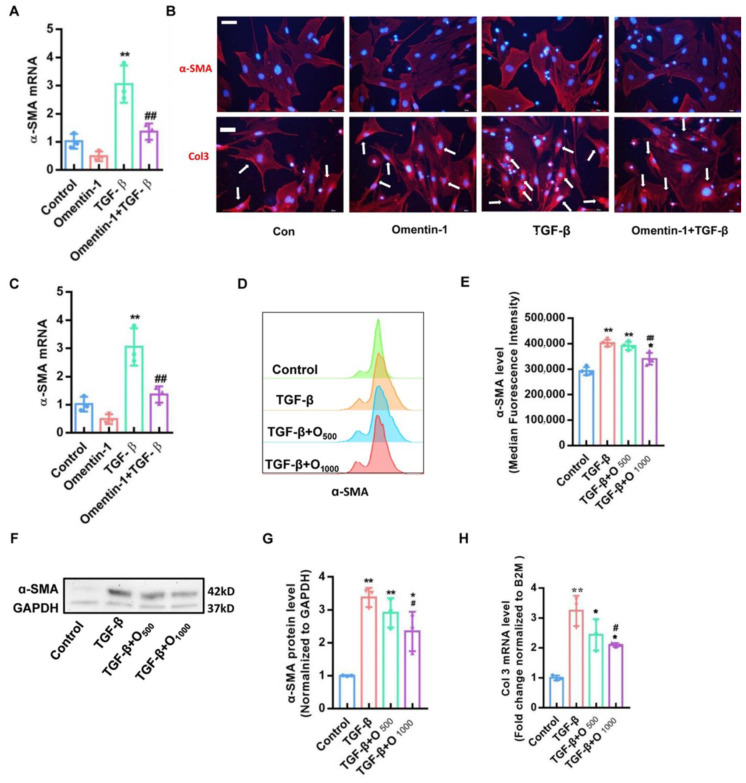
The effect of omentin-1 on TGF-β activated myofibroblast in vitro. (**A**) The mRNA level of α-SMA detected by qPCR in mouse lung fibroblasts (*n* = 3); (**B**) Immunofluorescence for α-SMA and Col3 in mouse lung fibroblasts (Bar = 25 μm, *n* = 3), White arrows indicate cells with α-SMA upregulation. (**C**) The mRNA level of α-SMA detected by qPCR in HLFs (*n* = 3); (**D**) α-SMA level in HLFs detected by Flow cytometry (*n* = 4); (**E**) Median Fluorescence intensity of α-SMA in HLFs. (**F**)The content of α-SMA in HLFs detected by Western blotting with cropping gel at the relative molecular size (*n* = 3); (**G**) Semi-quantitative analysis of α-SMA level evaluated by Western blotting; (**H**) The mRNA level of Col3 in HLFs detected by qPCR (*n* = 3). The data are presented as the mean ± SD. * *p* < 0.05, ** *p* < 0.01 vs. control group, # *p* < 0.05 vs. TGF-β group, ## *p* < 0.01 vs. TGF-β group.

To further investigate the role of omentin-1 on fibroblast activation, we tested the effect of recombinant omentin-1 on the transforming growth factor (TGF)-β1-induced fibroblast activation [36]. Pretreatment of omentin-1 (500 ng/mL) markedly attenuated the upregulation of α-SMA induced by TGF-β1 in primary mouse lung fibroblasts, as assessed by qPCR (Figure 5A) and immunofluorescence (Figure 5B). Moreover, the increased protein level of col 3 induce by TGF-β1 in primary mouse lung fibroblasts significantly decreased by omentin-1 as determined by immunofluorescence (Figure 5C). Similar findings were found in human lung fibroblasts (HLFs), as assessed by qPCR (Figure 5C), flow cytometry (Figure 5D,E) and Western blot (Figure 5F,G), showing that recombinant omentin-1 (1000 ng/mL) attenuated the level of α-SMA induced by TGF-β1. In addition, omentin-1 treatment reduced the mRNA level of col 3 in TGF-β1-treated HLFs (Figure 5H). Taken together, these results supported the role of omentin-1 in attenuating lung fibrosis by the inhibition of fibroblast activation.

### 3.5. Omentin-1 Attenuates Lung Fibrosis via Activation of AMPK Pathway

**Figure 6:**
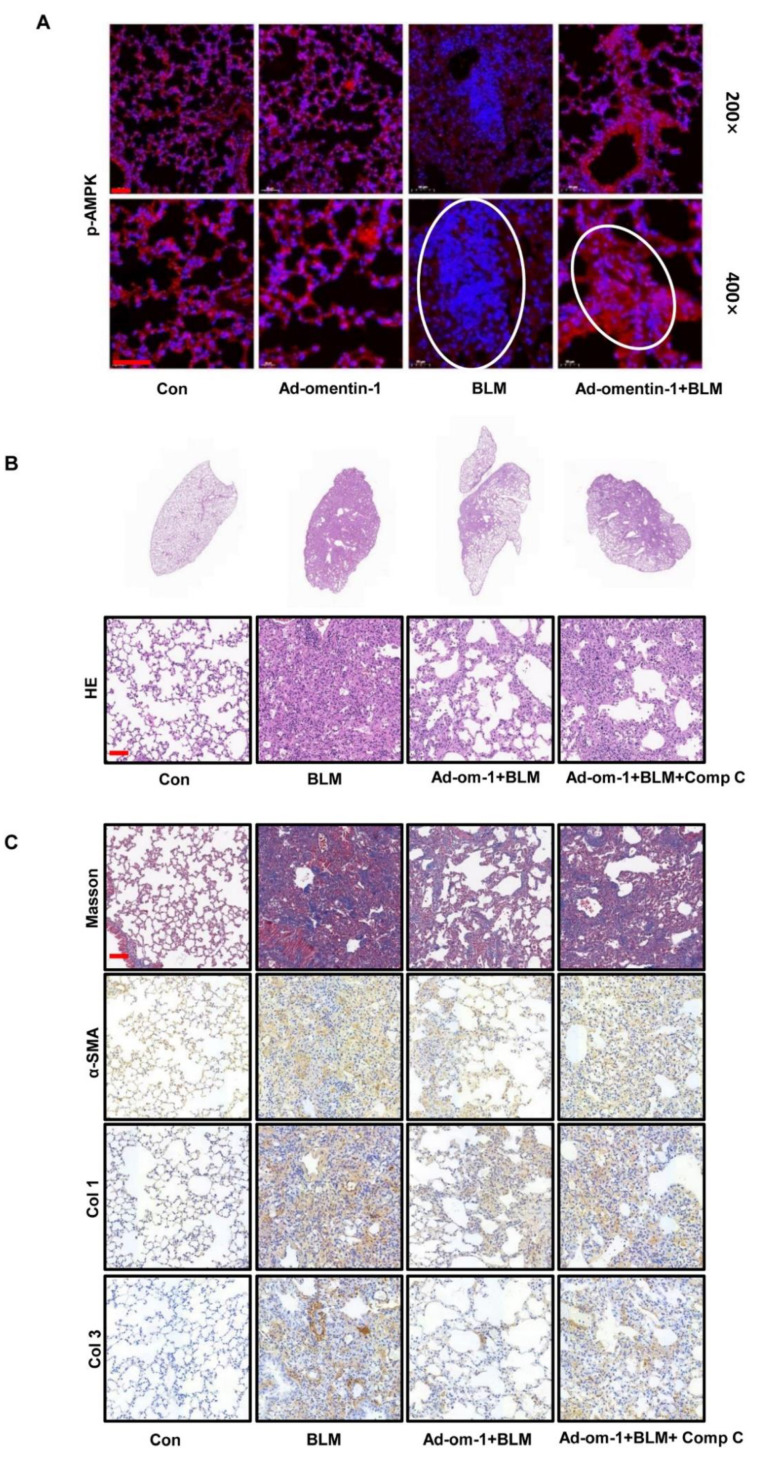
The effect of omentin-1 mediated by activation of AMPK pathway in vivo. (**A**) The level of p-AMPK in lung tissue detected by immunofluorescence after BLM challenge for 14 d and ad-omentin-1 preventative treatment (up: 200×, lower: 400×, bar = 50 μm, *n* = 3); (**B**) Representative micrographs of H&E staining of lung tissue (Bar = 50 μm, *n* = 3). (**C**) Representative micrographs of Masson trichrome staining, immunohistochemical staining of lung sections for α-SMA, Col1 and Col3 (Bar = 50 μm, *n* = 3).

To further explore the cellular mechanism of the anti-fibrosis effect of omentin-1, we detect the level of p-AMPK by immunofluorescence. Results showed that the level of p-AMPK significantly decreased in the BLM-injured lung tissue, especially in the fibrotic foci (marked by white circle, Figure 6A). The overexpression of omentin-1 significantly increased the level of p-AMPK in lung tissue in the therapeutic regimen (Figure 6A). These results indicate that omentin-1 can activate the AMPK pathway in lung tissues insulted by BLM. We used AMPK inhibitor, Compound C to further confirm that AMPK activation mediated the protective effect of omentin-1 on BLM-induced fibrosis. Our results showed that Comp C significantly attenuate the effect of omentin-1 on BLM-induced fibrosis, as showed by HE staining (Figure 6B), Masson staining, and immunochemistry of α-SMA, Col1 and Col3 (Figure 6C).

### 3.6. Omentin-1 Attenuates TGF-β-Induced Fibroblast Activation via AMPK Pathway

**Figure 7:**
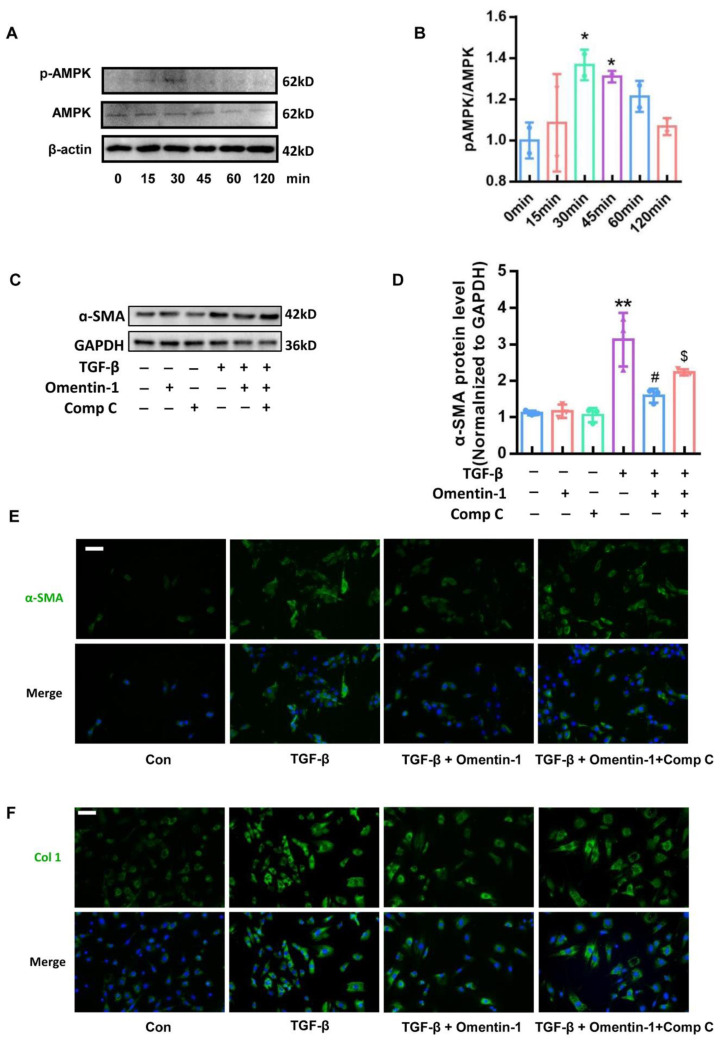
The effect of omentin-1 mediated by activation of AMPK pathway in vitro. (**A**) The content of p-AMPK, AMPK in mouse lung fibroblasts detected by Western blotting with cropping gel at the relative molecular size (*n* = 3). Cells were treated with omentin-1 (500 ng/mL) for 15, 30, 45, 60, 120 min; (**B**) Semi-quantitative analysis of p-AMPK/AMPK level evaluated by Western blotting with cropping gel at the relative molecular size. (**C**) The content of α-SMA in mouse lung fibroblasts detected by Western blotting (*n* = 3). Cells were pretreatment with Comp C (10 μM) for 1 h, then treated with omentin-1 (500 ng/mL) for 1h, then treat with TGF-β (10 ng/mL) for 48 h; (**D**) Semi-quantitative analysis of α-SMA level evaluated by Western blotting. (**E**) Immunofluorescence for α-SMA in mouse lung fibroblasts (Bar = 50 μm, *n* = 3). (**F**) Immunofluorescence for Col1 in mouse lung fibroblasts (Bar = 50 μm, *n* = 3). The data are presented as the mean ± SD. * *p* < 0.05, ** *p* < 0.01 vs. control group. # *p* < 0.05 vs. TGF-β group, $ *p* < 0.05 vs. TGF-β+Omentin-1 group.

To further confirm whether the effect of omentin-1 depends on the AMPK pathway in the fibroblast, we stimulated mouse fibroblast by TGF-β, with omentin-1 treatment in the absence of Comp C or not. Results showed that omentin-1 significantly increases the level of p-AMPK in the fibroblast at 30–45 min (Figure 7A,B). The effect of omentin-on TGF-β-induced α-SMA level (Figure 7C–E) and Col1 level (Figure 7F) was effectively blocked by Comp C.

## 4. Discussion

In the current study, we explored the role of omentin-1 in BLM-induced lung fibrosis and evaluated its impact on fibroblast activation. We observed dynamic changes in the levels of omentin-1 mRNA and protein during the fibrotic process, which especially increased at 21 days after the BLM challenge. We provided the first evidence that omentin-1 depletion aggravated BLM-induced lung fibrosis, which was reflected in increased weight loss, more severe destruction of the lung tissue structure, much higher collagen deposition, as well as a higher level of α-SMA in lung tissue. Moreover, adenovirus-mediated omentin-1 overexpression potently inhibited BLM-induced pulmonary fibrosis, both in preventive and therapeutic regimens. Omentin-1 overexpression significantly attenuated BLM-induced pathological alterations, including the increase in the levels of Collagen type 1 and 3, hydroxyproline, and α-SMA. Furthermore, omentin-1 overexpression inhibited fibroblast activation in vivo, and recombinant omentin-1 treatment mitigated TGF-β1-induced myofibroblast differentiation and collagen deposition in vitro via activation of the AMPK pathway. Taken together, our results demonstrated that omentin-1 protected mice from BLM-induced pulmonary fibrosis via direct anti-fibrosis effects, partially by inhibiting fibroblast activation by activating the AMPK pathway.

BLM-induced pulmonary fibrosis is a well-established model of pulmonary fibrosis [37]. This fibrosis model is characterized by acute inflammation at day 7, established fibrosis at day 14, and peak of fibrosis at days 21–28 [27]. Numerous anti-inflammatory agents inhibit BLM-induced fibrosis in animal models, but none of them has shown comparable effects in human IPF clinical trials [38]. Thus, when evaluating new therapeutic strategies, it is crucial to distinguish between anti-inflammatory and anti-fibrotic effects. Our previous study showed that omentin-1 overexpression protects against BLM-induced acute inflammation by inhibiting macrophage activation [26]. Thus, to selectively evaluate the anti-inflammatory and antifibrotic properties of omentin-1, we here investigated the effects of adenovirus-mediated omentin-1 overexpression 3 days before the BLM challenge, at the beginning of the inflammatory response (preventive treatment), as well as 11 days after BLM challenge, when most of the inflammatory response was resolved (therapeutic treatment). Our results showed that omentin-1 protected BLM-induced fibrosis in both the preventive and the therapeutic settings. Accordingly, omentin-1-deficient mice showed aggravated BLM-induced lung fibrosis. We hypothesize that omentin-1 is an endogenous adipokine that attenuates lung fibrosis, similarly to adiponectin, another adipokine synthesized and secreted by adipocytes [39]. As an endogenous anti-fibrosis agent, adiponectin exerts a protective effect against liver, renal, and pulmonary fibrosis by inhibiting the activation of myofibroblasts [40,41].

Myofibroblasts are scar-forming cells and key effectors in fibrosis, and are responsible for the excessive synthesis, deposition, and remodeling of extracellular matrix proteins in lung fibrosis [5]. Preventing myofibroblast activation [17,42], inducing myofibroblast apoptosis [17,43] or promoting myofibroblast dedifferentiation [32,33] are necessary to achieve fibrosis resolution, namely the elimination of ECM-producing cells. In our study, we showed that omentin-1 prevented fibroblast activation into myofibroblasts, both in vivo and in vitro.

**Figure 8:**
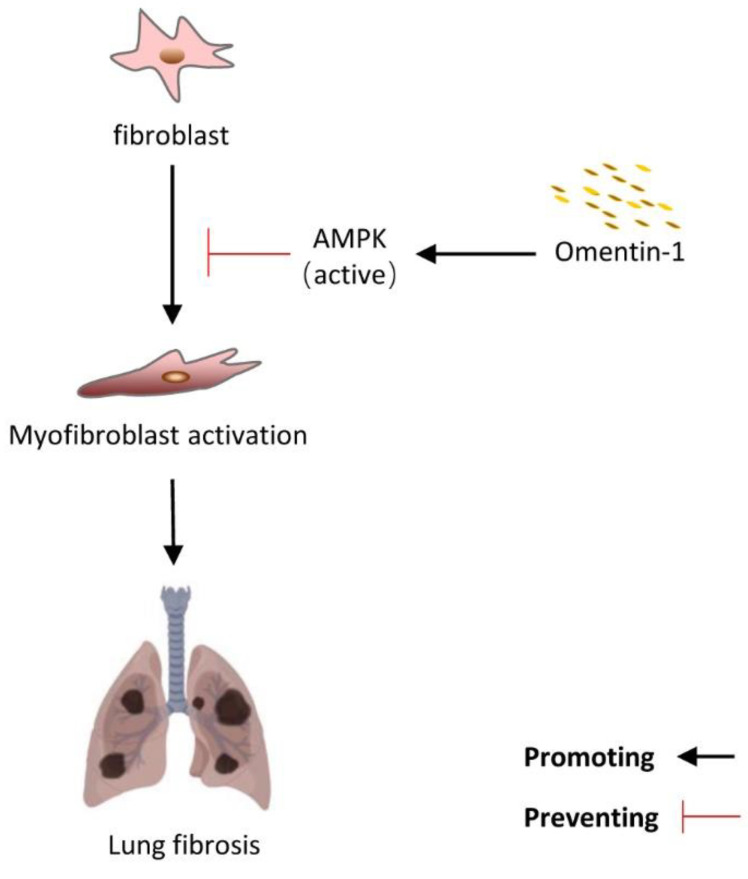
The summary of the effect of omentin-1 in lung fibrosis.

AMPK is an important regulator in controlling energy metabolism, and has become an important strategic cellular target for lung fibrosis. Several researches showed that promoting activation of AMPK in fibroblasts and lung fibrosis tissue significantly alleviated lung fibrosis [19,20,21]. Increasing evidence has indicated that omentin-1 can activate AMPK and alleviate myocardial ischemic injury [44] and high glucose-induced endothelial dysfunction [25]. In our study, omentin-1 treatment could significantly increase the p-AMPK level and attenuated lung fibrosis in lung tissue injured by BLM, which can be blocked by Comp C (inhibitor of AMPK). To further evaluated the cellular mechanism, cells were pretreatment with Comp C. We found that Comp C significantly blocked the effect of omentin-1 on decreasing α-SMA level induced by TGF-β. Collectively, our results indicate that AMPK is one of the important signal nodes that can mediate the inhibition effect of omentin-1 on fibroblast activation and lung fibrosis (Figure 8).

## 5. Conclusions

In summary, omentin-1 protected mice from BLM-induced pulmonary fibrosis, partially by preventing fibroblast activation via activating the AMPK pathway. Our results suggest that omentin-1 is an endogenous anti-fibrosis adipokine and could be a potential therapeutic strategy for lung fibrosis.

## Figures and Tables

**Table 1:** Sequences of specific primers (mouse and human) used in this study.

Gene	Forward Primer Sequence (5 to 3)	Reverse Primer Sequence (5 to 3)	Length (bp)
*M-GAPDH*	GAAGGTGGTGAAGCAGGCATCT	CGGCATCGAAGGTGGAAGAGTG	116
*M-α-SMA*	CTTCGCTGGTGATGATGCTC	GTTGGTGATGATGCCGTGTT	175
*M-Omentin-1*	AGTGCAGCTGAAGAGAACCT	ACTTCCCACGCATGTTGTTC	229
*M-Col1a1*	GAGCGGAGAGTACTGGATCG	GCTTCTTTTCCTTGGGGTTC	158
*M-Col3*	GCACAGCAGTCCAACGTAGA	TCTCCAAATGGGATCTCTGG	185
*H-B2M*	TTTCATCCATCCGACATTGA	CCTCCATGATGCTGCTTACA	228
*H-α-SMA*	TTCAATGTCCCAGCCATGTA	GAAGGAATAGCCACGCTCAG	222
*H-Col 1a1*	CCAAATCTGTCTCCCCAGAA	TCAAAAACGAAGGGGAGATG	214
*H-Col3*	TACGGCAATCCTGAACTTCC	GTGTGTTTCGTGCAACCATC	245

## Data Availability

Please contact corresponding author for data accessibility.

## Abbreviations

ad-omentin-1	Adenovirus omentin-1
AMPK	AMP-activated protein kinase
ATP	Adenosine Triphosphate
α-SMA	α -smooth muscle actin
BLM	Bleomycin
B2M	β2 microglobulin
CT	computed tomography
Comp C	Compound C
ECM	extracellular matrix
GAPDH	Glyceraldehyde-3-phosphate dehydrogenase
H&E	Hematoxylin and eosin
HYP	hydroxyproline
IPF	idiopathic pulmonary fibrosis
SARS-CoV-2	Severe acute respiratory syndrome coronavirus 2
TGF-β1	transforming growth factor beta 1
omentin-1^−/−^	omentin-1-deficient
p-AMPK	phosphorylation of AMPK
qPCR	quantitative Polymerase Chain Reaction
WB	Western blotting
WT	Wild Type
